# Diagnostic performance of regional cerebral blood flow images derived from dynamic PIB scans in Alzheimer’s disease

**DOI:** 10.1186/s13550-019-0528-3

**Published:** 2019-07-04

**Authors:** Débora E. Peretti, David Vállez García, Fransje E. Reesink, Janine Doorduin, Bauke M. de Jong, Peter P. De Deyn, Rudi A. J. O. Dierckx, Ronald Boellaard

**Affiliations:** 1 Department of Nuclear Medicine and Molecular Imaging, University Medical Center Groningen, University of Groningen, Hanzeplein 1, 9713 GZ Groningen, The Netherlands; 2 Department of Neurology, Alzheimer Centrum Groningen, University Medical Center Groningen, University of Groningen, Hanzeplein 1, 9713 GZ Groningen, The Netherlands; 3 Laboratory of Neurochemistry and Behaviour, Institute Born-Bunge, University of Antwerp, Universiteitsplein 1, 2610 Antwerpen, Belgium

**Keywords:** Alzheimer’s disease, PIB, Relative cerebral blood flow, PALZ

## Abstract

**Background:**

In clinical practice, visual assessment of glucose metabolism images is often used for the diagnosis of Alzheimer’s disease (AD) through 2-[^18^F]-fluoro-2-deoxy-d-glucose (FDG) positron emission tomography (PET) scans. However, visual assessment of the characteristic AD hypometabolic pattern relies on the expertise of the reader. Therefore, user-independent pipelines are preferred to evaluate the images and to classify the subjects. Moreover, glucose consumption is highly correlated with cerebral perfusion. Regional cerebral blood flow (rCBF) images can be derived from dynamic ^11^C-labelled Pittsburgh Compound B PET scans, which are also used for the assessment of the deposition of amyloid-β plaques on the brain, a fundamental characteristic of AD. The aim of this study was to explore whether these rCBF PIB images could be used for diagnostic purposes through the PMOD Alzheimer’s Discrimination Tool.

**Results:**

Both tracer relative cerebral flow (*R*_1_) and early PIB (ePIB) (20–130 s) uptake presented a good correlation when compared to FDG standardized uptake value ratio (SUVR), while ePIB (1–8 min) showed a worse correlation. All receiver operating characteristic curves exhibited a similar shape, with high area under the curve values, and no statistically significant differences were found between curves. However, *R*_1_ and ePIB (1–8 min) had the highest sensitivity, while FDG SUVR had the highest specificity.

**Conclusion:**

rCBF images were suggested to be a good surrogate for FDG scans for diagnostic purposes considering an adjusted threshold value.

**Electronic supplementary material:**

The online version of this article (10.1186/s13550-019-0528-3) contains supplementary material, which is available to authorized users.

## Background

Positron emission tomography (PET) imaging improves the diagnosis of Alzheimer’s disease (AD) due to the broad range of functional processes it assesses [1]. One of the most common radiotracers for PET scans, both in the clinic and in research, is 2-[^18^F]-fluoro-2-deoxy-d-glucose (FDG). This radiotracer evaluates the metabolism in the brain by measuring glucose consumption, allowing for the recognition of specific disease patterns. AD is characterized by a hypometabolic pattern that includes regions such as the precuneus, posterior cingulate cortex, posterior temporoparietal cortex, and medial temporal lobe [2]. The identification of the hypometabolic pattern caused by the disease is of great importance for clinicians during the diagnostic process. Another advantage of using FDG-PET is that it is sensitive to changes in the early stages of the disease, even in patients without clinical symptoms of dementia [3–5]. However, visual reading of the FDG-PET images relies on the experience of the reader [2, 6]. Therefore, different methods of user-independent analyses have been developed to assist in the interpretation of the scans [7–9].

Previous studies have shown a link between glucose consumption and regional cerebral blood flow (rCBF): blood delivery across the brain increases with metabolic demand [10, 11]. This link might allow the use of rCBF images for the classification of AD patients since regions that have the glucose consumption affected might also be hypoperfused. These rCBF images can be derived from standardized uptake value ratio (SUVR) of radiotracers that measure the flow in the brain, such as ^15^O-Water [12], the weighted average of the initial frames of a dynamic scan [13–15], or through pharmacokinetic modelling [16–18].

A radiotracer that is commonly used in AD trials and in the clinic is ^11^C-labeled Pittsburgh Compound B (PIB). PET scans with PIB allow the clinician to assess the deposition of amyloid-β (Aβ) plaques in the brain. Therefore, FDG and PIB images provide complementary information that improve the diagnosis of AD [1, 19]. Yet, dual-tracer studies can be expensive and increase patient discomfort and exposure to radiation [17, 20]. Hence, the use of a single tracer to assess both Aβ deposits and the hypometabolism pattern in the brain at the same time would be ideal. In this respect, using both the tracer’s binding potential and parametric images of relative tracer flow (*R*_1_) might provide such complementary image information concerning Aβ deposition and rCBF, respectively.

Since PIB possesses high lipophilicity [21], it meets the prerequisite to provide rCBF images that might be a good surrogate for FDG. This hypothesis has already been explored in previous studies, which compared FDG scans to PIB images generated through pharmacokinetic modelling [16–18, 22], and a time-weighted average of the first frames of a dynamic PIB scan [13, 22–25].

A commonly known tool for the automated discrimination of AD patients is PMOD Alzheimer’s Discrimination Tool (PALZ). The user provides PALZ with the FDG images of a subject, which is compared to a database of healthy controls. PALZ estimates how different is the metabolism pattern of the provided image from a group of typical healthy subjects [6], and gives a score that helps to determine whether the subject presents an abnormal scan. Although this automated discrimination tool was designed for FDG, rCBF images might provide similar results due to the high correlation between images.

The aim of this study was to explore whether rCBF images, derived from dynamic PIB scans, could be used for the diagnosis of AD using the PALZ tool from PMOD. To this end, *R*_1_ and summed early frame images were generated and used as input images in PALZ. The results were then compared to the results from the FDG scans. Correlations between scores and new thresholds for classifying AD patients were drawn for each method.

## Material and methods

### Subjects

A cohort of fifty-two subjects was drawn from a larger ongoing study at the memory clinic of the University Medical Center Groningen (UMCG), Groningen, The Netherlands. All subjects gave their written informed consent to participate in the study, which was approved by the Medical Ethical Committee of the UMCG (2014/320). The study was conducted in agreement with the Declaration of Helsinki and subsequent revisions.

The subjects were first diagnosed by consensus of a multidisciplinary team based on clinical assessment following the guidelines of the National Institute on Aging Alzheimer’s Association criteria (NIA-AA) [26] for the AD patients, and on the Petersen criteria [27] for the MCI patients. Healthy subjects presented no cognitive complaints and a mini-mental state exam score higher than 28. Then, all subjects underwent two PET scans and a T1-3D magnetic resonance imaging (MRI). After this, clinical diagnoses were reconsidered under the National Institute on Aging and the Alzheimer’s Association Research Framework [28]. Subjects were then reclassified as AD, MCI+ (mild cognitive impairment with Aβ deposition), MCI− (mild cognitive impairment or other dementia without Aβ deposition), or healthy controls (HC). Positivity or negativity regarding Aβ deposition was done by consensus of visual inspection by experts. A summary of the demographic characteristics is shown in Table 1.

**Table 1:** Demographic characteristics of subjects

	AD (*n* = 15)	MCI+ (*n* = 11)	MCI− (*n* = 10)	HC (*n* = 16)
Sex				
Male	9	7	8	11
Female	6	4	2	5
Age (years)	65 ± 8	65 ± 5	67 ± 9	69 ± 5
MMSE score	25 ± 3	27 ± 2	24 ± 7	30 ± 1

### PET acquisition

All subjects underwent a static FDG-PET and a dynamic PIB-PET examination. Scans were performed with either a Siemens Biograph 40mCT or 64mCT scanner (Siemens Medical Solution, USA). Since both systems are of the same vendor and of the same generation, the acquisition and reconstruction protocols were harmonized, and the calibration of the systems was equally done; no difference between data provided by the different scanners was expected. Nonetheless, a *t* test comparing the results provided by the different scanners showed that there were no statistically significant differences between them. Patients were in standard resting conditions with eyes closed during the scans. The radiotracers were synthesized at the radiopharmacy facility at the Nuclear Medicine and Molecular Imaging department at the UMCG, according to Good Manufacturing Practice, and were administered via venous cannula. The subjects had both scans performed on the same month, with the FDG taking place at least 90 min after PIB injection, with the exception of five subjects, who had a delay of up to 4 months between scans.

The dynamic PIB-PET acquisitions started 10 s before tracer injection (375 ± 50 MBq) and lasted at least 60 min (frames: 7 × 10 s, 3 × 30 s, 2 × 60 s, 2 × 120 s, 2 × 180 s, 5 × 300 s, and 2 × 600 s). The static FDG-PET scans were acquired 30 min after injection (203 ± 8) and lasted for 20 min. All subjects were fasted for at least 6 h before injection, and glucose levels in plasma were measured before the scan, and the PET scan was only performed if glucose levels were lower than 7 mmol/l [29]. All PET images were reconstructed from list-mode data using 3D OSEM (3 iterations and 24 subsets), point spread function correction, and time-of-flight. The resulting images had a matrix of 400 × 400 × 111, with isotropic 2 mm voxels, and smoothed 2-mm Gaussian filter at full width and half maximum (FWHM).

### Image processing

The PMOD software package (version 3.8; PMOD Technologies LLC) was used for image registration and data analysis. The MRI scans were normalized to the Montreal Neurological Institute (MNI) space using tissue probability maps [30]. The PIB-PET images were first corrected for motion (in case of any) using the averaged first 13 frames as reference and then aligned to the individual MRI. The Hammers atlas [31] was used to draw the volume of interest (VOI) of the grey matter from the cerebellum. All PET images were smoothed using a 6-mm Gaussian filter at FWHM, and all voxels outside of the brain were masked.

The *R*_1_ parametric images were generated by pharmacokinetic modelling on a voxel level of the PIB-PET scans in individual space. The simplified reference tissue model 2 (SRTM2) [32] was chosen for this analysis [33], with the grey matter from the cerebellum as the reference tissue [34–37]. A first estimate of the binding potential (*BP*_ND_) was done using the simplified reference tissue (SRTM) [38], so the efflux parameter of the reference region (*k*_2_′) could be fixed. This parameter was taken as the median value from all voxels with a *BP*_ND_ higher than 0.05. Then, SRTM2 was applied with a restriction on the range of the apparent efflux rate constant values, with a minimum of 0.01 and a maximum of 0.03, and 80 basis functions to generate the final *R*_1_ parametric maps.

The early-stage PIB (ePIB) distribution images were generated using the time-weighted average of the frames corresponding to the intervals of 20 to 130 s and 1 to 8 min. These intervals were chosen because previous studies have found that 20 to 130 s was the best interval to discriminate between patients and healthy subjects [22], and 1 to 8 min have shown the best correlation with FDG scans [24]. Then, the standardized uptake value ratios (SUVR) were calculated by dividing each voxel of the image by the ratio of the injected dose and body weight of the subject and normalizing to the average value of the reference region (i.e. grey matter of the cerebellum).

To compare with the FDG-PET images, the FDG SUVR images were generated in the same manner as the ePIB SUVR, also using the grey matter of the cerebellum as the reference region.

For each subject, an FDG SUVR, *R*_1_, ePIB (20–130 s), and ePIB (1–8 min) were generated and evaluated by PALZ (v3.9, PMOD Technologies LLC), which gave a PET_SCORE_ per image for each method according to how much the regions typically affected by AD deviated from what is expected of a healthy person. An overview of the steps taken for analysing the images is provided in Additional file 1.

### Statistical analysis

An ANOVA per method was performed to check if the groups presented significantly different PET_SCORES_ for each method. Then, a pairwise *t* test was done to compare the significance between groups within methods. For this test, the *p* values were adjusted for multiple comparisons using the Holm method [39].

A general linear model was used to explore the relationship between the PET_SCORES_ of each PIB-derived image (independent variable) and the FDG SUVR (dependent variable) for all subjects. A *p* value of 0.05 was used as a significance threshold for all analyses. No correction for multiple comparisons was made.

A Bland-Altman plot was made to evaluate the agreement between the PET_SCORE_ measured by PIB-derived methods and FDG SUVR. The difference between scores was plotted against the FDG SUVR PET_SCORES_, since these scores were considered the reference values [40]. Furthermore, linear regressions were made to assess the bias of each rCBF measure compared to the FDG SUVR PET_SCORES_.

Receiver-operating characteristic (ROC) curves were plotted to estimate the sensitivity and specificity of each method using Youden’s method [41], using only the PET_SCORE_ from the AD and HC groups, since PALZ was developed to differentiate AD from healthy subjects, and not between different types of dementia. DeLong’s test was applied to find if there was a correlation between the rCBF and FDG SUVR curves [42]. All results were analysed using RStudio (version 1.1.456, R version 3.5.1 [43]).

## Results

### PET_SCORES_

**Fig. 1:**
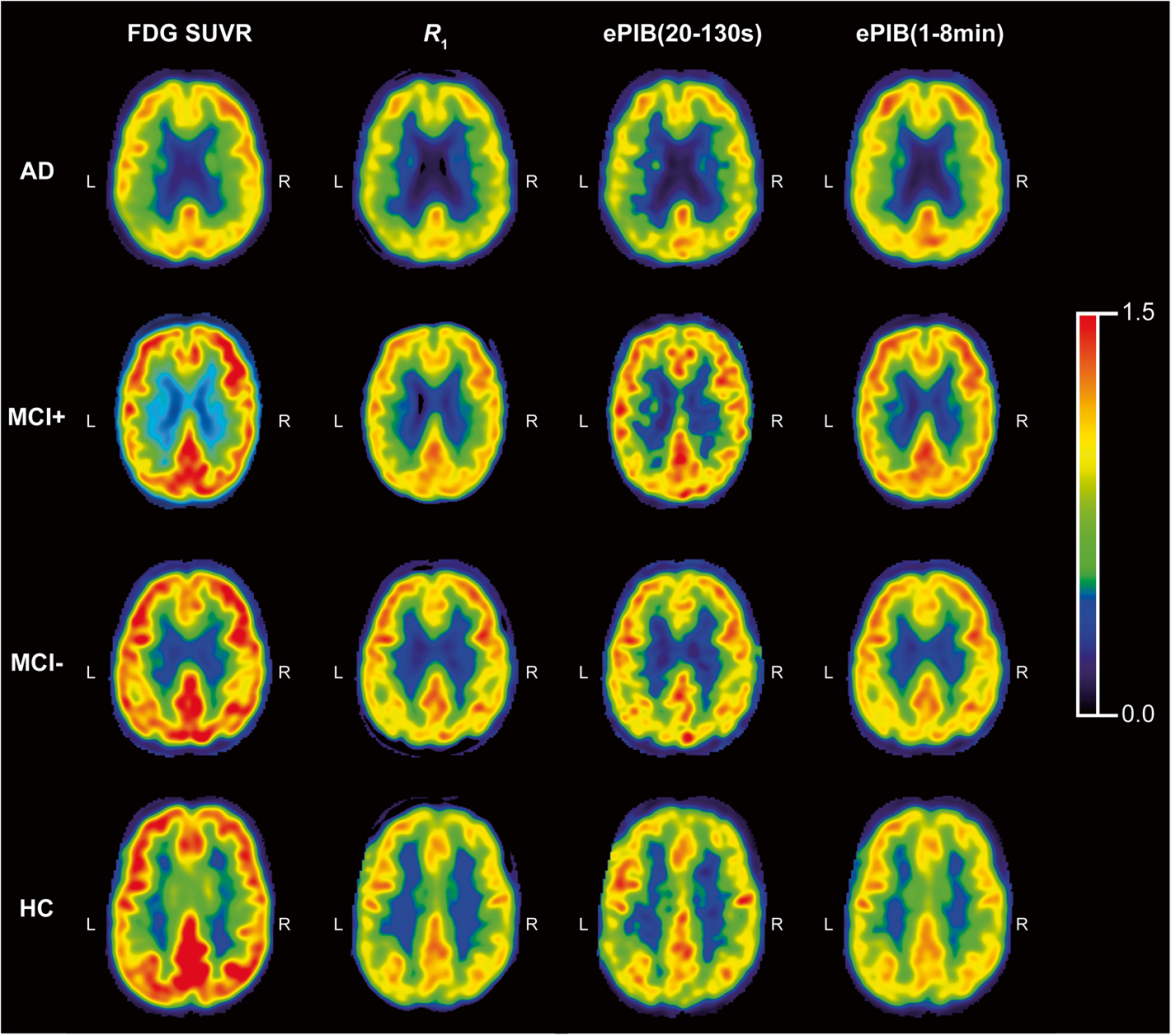
Representative studies. Transaxial slices of the brain are shown. From left to right: FDG SUVR images, *R*_1_ parametric maps, ePIB (20–130 s), and ePIB (1–8 min) images. On the first row, images from an AD patient; on the second, an MCI+ subject; on the third, an MCI− subject; and at the bottom, an HC subject. All colour scales are adjusted to the same range

In general terms, the FDG SUVR images were in agreement with the pattern expected from the literature (Fig. 1), showing a hypometabolic pattern for the AD group, while the HC subjects presented no abnormal cortical uptake of the tracers. The resemblance between the *R*_1_ and ePIB when compared to the FDG SUVR was also notable, with similar AD patterns of decreased flow on the parietal lobe, for example.

**Fig. 2:**
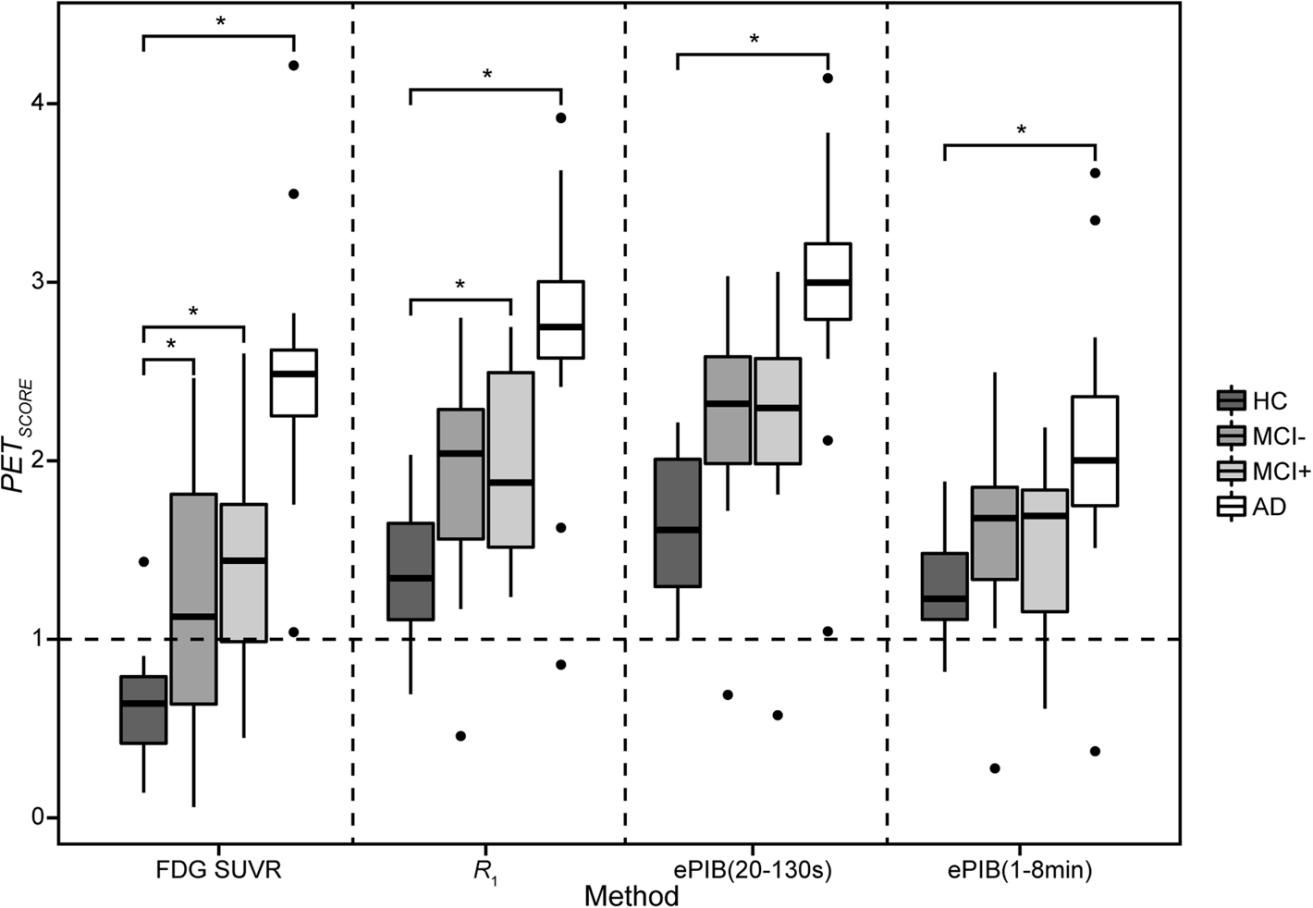
Distribution of PET_SCORES_ per method. Distribution of subjects’ PET_SCORES_ from FDG SUVR, *R*_1_, ePIB (20–130 s), and ePIB (1–8 min) respectively from left to right. Darkest grey boxes represent data from the AD group; dark grey represents MCI+ subjects; light grey represents MCI−; and white represents HC. A dashed line at PET_SCORE_ = 1 represents the threshold from PALZ for the classification of AD patients. The stars represent the differences between the groups that are statistically significant

The distribution of the PET_SCORES_ for each method for all subjects is shown in Fig. 2. In general, all methods presented a statistically significant difference between groups (*p* < 0.05). All methods were also able to differentiate between the AD and HC groups, but none of them was capable of distinguishing between MCI+ and MCI−. While the FDG SUVR was able to show a statistically significant difference between the HC and both MCI+ and MCI− groups, of the rCBF methods, only *R*_1_ presented a significant difference between the HC and MCI+ groups. Means, standard deviations, and range of the scores of all groups for all methods can be seen in Additional file 2: Table S2.

### Correlation of scores from FDG SUVR, *R*_1_, and ePIB

**Fig. 3:**
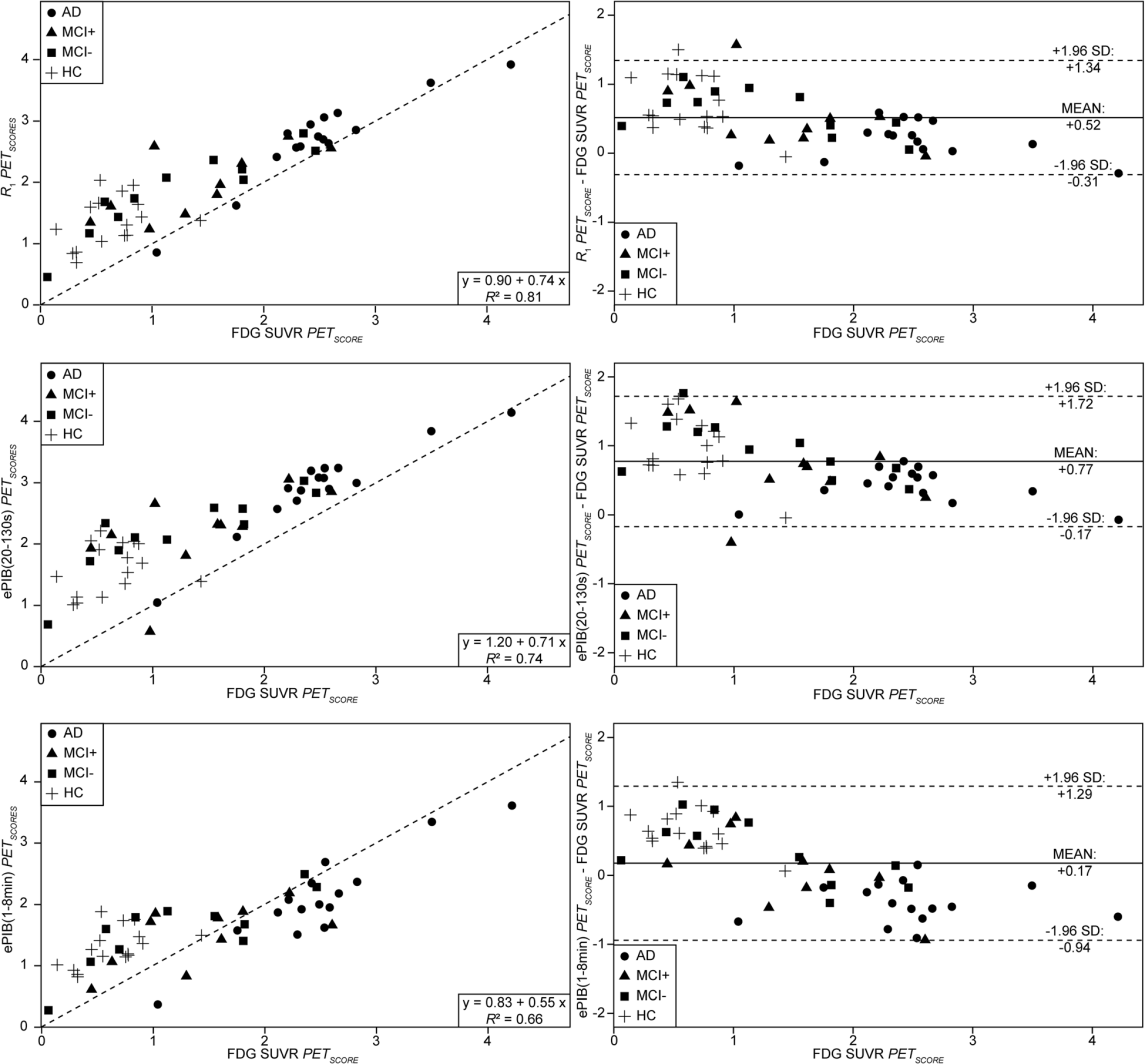
Scatter and Bland-Altman plots. Scatter plots (first column) showing PET_SCORES_ from *R*_1_ parametric maps (top), ePIB (20–130 s) (middle), and ePIB (1–8 min) images (bottom) (*y*-axis), and from FDG SUVR (*x*-axis). The dashed lines display the identity line. Results of the linear regression are given in boxes at the bottom right corner. Bland-Altman plots (second column) showing the difference between the PET_SCORES_ provided by *R*_1_ (top), ePIB (20–130 s) (middle), and ePIB (1–8 min) and FDG SUVR. The full line is at the mean difference value for all scores, and the dashed lines delimit the 95% agreement interval (at mean ± 1.96 × standard deviation). Data are arranged according to subject group: circles represent the AD, triangles the MCI+, squares the MCI−, and cross the HC group

The scatter plots of the scores given to the FDG SUVR images suggest a high correlation with the images provided by the rCBF images (Fig. 3). *R*_1_ presented a correlation of 0.90 with the FDG SUVR, which was the highest correlation across all rCBF methods. The scores from FDG SUVR were highly predictive of the ones from *R*_1_, accounting for 81% of variability (*R*^*2*^ = 0.81, *p* < 0.001, intercept = 0.90, slope = 0.74). The scores from ePIB (20–130 s) also presented a high correlation as compared to the ones from FDG SUVR (0.87), but the predictability of the method was lower, 74% (*R*^*2*^ = 0.74, *p* < 0.001, intercept = 1.20, slope = 0.71). While ePIB (1–8 min) PET_SCORES_ also presented a high correlation as compared to the ones from FDG SUVR, of 0.82, this method was not as predictive as the other two, accounting for 66% of the variability only (*R*^2^ = 0.66, *p* < 0.001, intercept = 0.83, slope = 0.55).

### Bias assessment

**Fig. 4:**
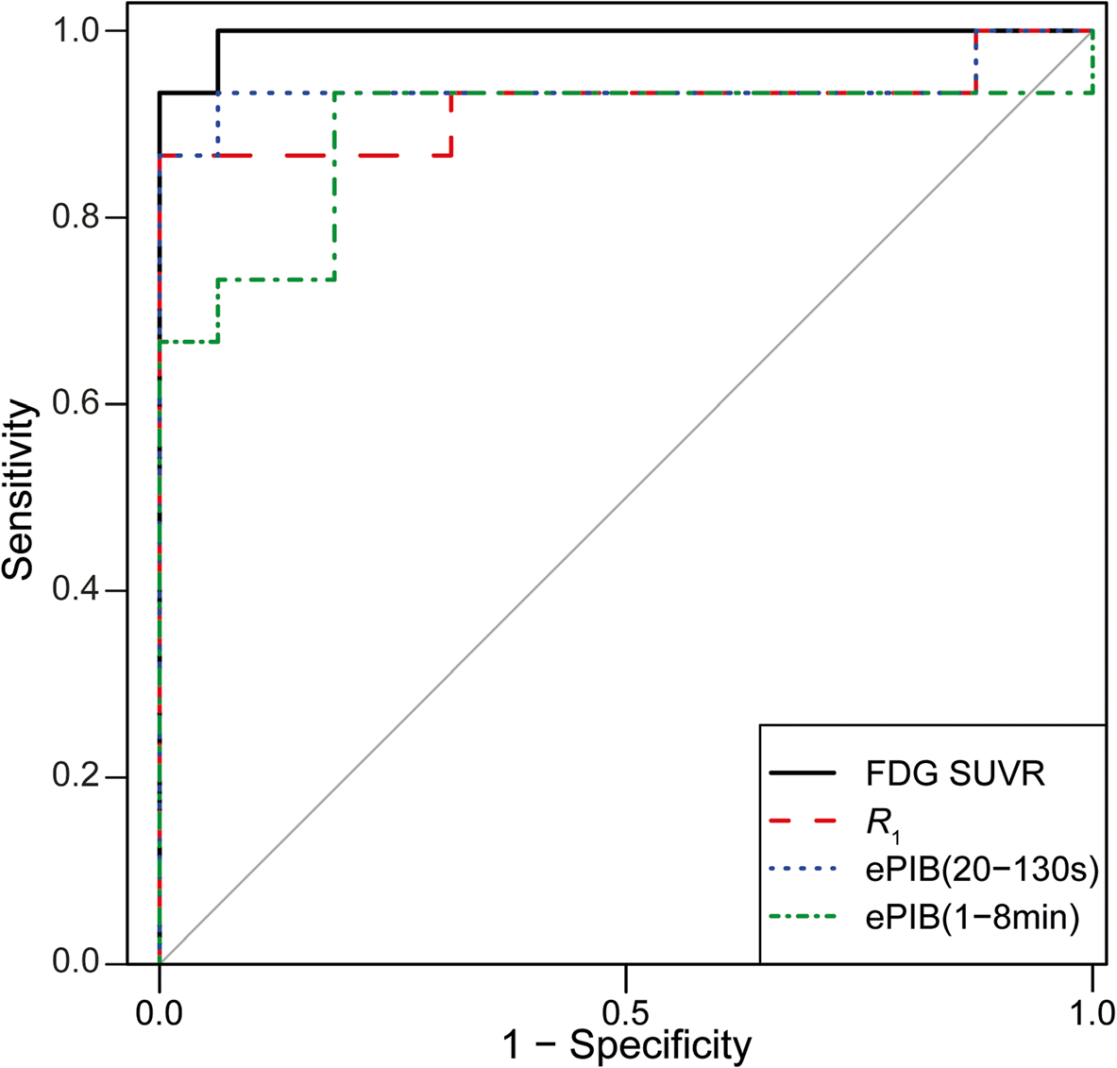
ROC plots*.* ROC plot with the curves of FDG SUVR (solid line), *R*_*1*_ (dashed line), ePIB (20–130 s) (dotted line), and ePIB (1–8 min) (dot dashed line)

The bias found between FDG SUVR and rCBF PET_SCORES_ was monophasic for the *R*_1_ and ePIB (20–130 s) methods, meaning that, in general, they overestimated the PET_SCORES_, of 26% for the *R*_1_ (slope = -0.26, intercept = 0.9), and 29% for the ePIB (20–130 s) (slope = -0.29, intercept = 1.20). Meanwhile, the ePIB (1-8 min) method presented a biphasic relationship (slope = -0.45, intercept = 0.83): it overestimates the PET_SCORES_ of the HC group by approximately 50% while underestimating the AD group by nearly 19%. In summary, the *R*_1_ method presented the smallest bias of all methods and this bias was larger for the HC subjects than for the AD patients (Fig. 4) [44, 45].

### ROC curves

With the ROC curves (Fig. 4), it was possible to find a new PET_SCORE_ threshold for classifying the subjects as AD or HC for each of the rCBF methods. The optimal threshold for the best differentiation of the groups was of 2.22 for the *R*_1_ method, with a sensitivity of 0.87 and a specificity of 1. The second highest threshold was 2.08, from the ePIB (20–130 s) method, with a sensitivity of 0.93 and a specificity of 0.94. The ePIB (1–8 min) method resulted in a threshold of 1.50 for differentiating the groups, with a sensitivity of 0.93 and a specificity of 0.81. The ROC curves also showed that the area under the curve was high for all methods, the highest being for the FDG SUVR (0.99), followed by ePIB (20–130 s) (0.94), *R*_1_ (0.92), and ePIB (1–8 min) (0.89). No statistically significant differences were found between the rCBF and FDG SUVR curves.

## Discussion

The aim of this study was to use rCBF images derived from PIB-PET scans as a surrogate for FDG through an automated discrimination tool. The tool used in this work was PALZ, from PMOD Technologies. PALZ gives the images a PET_SCORE_ and classifies the subjects as AD or not based on a threshold of 1. This tool uses FDG-PET scans for the diagnosis of the subjects, but since metabolism and blood flow in the brain are highly correlated [10], this tool might also be used to distinguish between groups using rCBF images. Furthermore, the most recent guidelines for AD studies require Aβ imaging for the diagnosis of AD [46]. Therefore, the use of PIB-derived rCBF images in place of FDG scans, since PIB is already used for Aβ imaging, might be of advantage since it reduces costs and patient discomfort and exposure to radiation.

The distribution of the PET_SCORES_ showed a clear distinction between methods, with the FDG SUVR PET_SCORES_ being smaller than the ones from rCBF images, especially for the HC group (Fig. 2). It can also be seen that FDG SUVR, *R*_1_, and ePIB (20–130 s) PET_SCORES_ presented a clearer distinction between groups than ePIB (1–8 min). This suggests that ePIB (1–8 min) might not be an optimal method to diagnose patients, as it has already been observed in a previous study [22]. No significant distinction was found between MCI+ and MCI− groups, which was expected since PALZ was not developed to differentiate between diseases, but only to distinguish the AD patients from the HC subjects.

Overall, the high correlation between PET_SCORES_ provided by different methods indicates that rCBF images might be a good surrogate to FDG SUVR images. However, the slopes and intercepts of the linear regressions suggest that the threshold should be adjusted depending on the method used to generate the images. Furthermore, the bias between scores was different depending on the group, with a smaller bias for the AD patients than for the HC subjects. This difference might be related to the fact that rCBF images have a better correlation with FDG SUVR in patients with more binding of PIB than in subjects with no specific binding in cortical matter, which is the case for the HC subjects [22].

Moreover, in a comparison of each of the rCBF methods individually with FDG SUVR, *R*_1_ seemed to outperform both ePIB methods. The higher correlation and small bias from this method lead to the conclusion that the *R*_1_ images might be the method of preference to substitute FDG-PET scans when an automated tool to differentiate subjects is used, as was suggested by previous studies [22, 23].

Additionally, due to their high sensitivity and the fact that its ROC curve was not significantly different from those seen with FDG SUVR, rCBF images with an adjusted threshold are able to make a satisfactory distinction between groups for diagnostic purposes. The different thresholds found for each rCBF method suggest that, although they measure the same parameter, they do not yield the same results. This might be due to the fact that ePIB methods might be affected by some tracer binding already early after tracer administration, while *R*_1_ is a measure of only flow relative to that of the cerebellum. Furthermore, it is important to mention that the same data was used to estimate the new threshold for classification of subjects and to estimate its performance, which might have led to overfitting. Therefore, the area under the ROC curve may provide a better performance estimate than the sensitivity and specificity results.

Although the results presented in the previous section show a good correlation between rCBF and FDG SUVR, these results should be taken with caution. PALZ pipeline (Additional file 2: Table S1) includes comparing the input image with a database of FDG scans of healthy volunteers, which might have declined the precision of the resulting rCBF scores. Therefore, even though the PALZ works for rCBF images given an adjusted threshold, the classification of the images could be improved by providing a tracer-specific database of HC subjects. Furthermore, the introduction of the MCI+ and MCI− groups might have affected the results. This is due to the fact that PALZ is designed and validated only for the differentiation of AD patients from HC, as mentioned above. But previous studies have shown that PALZ is more sensitive to disease progression than are clinical tests in the MCI group [2, 47]. For this reason, the MCI group was also included in this analysis. Moreover, there is still a need of longitudinal studies to assess changes in *R*_1_ with the disease progression, since *R*_1_ has shown not to be as sensitive as FDG in scenarios where small effect sizes are relevant. Furthermore, a limited number of subjects for setting the new threshold were used in this study; an increased number of patients could improve the accuracy of the threshold. In addition, the diagnosis of the patients was done based on the visual assessment of the images, which might have introduced some bias in the performance of PALZ, overestimating the performance of the tool.

## Conclusion

The present study had the goal of using PIB-derived rCBF images as a surrogate for FDG-PET scans to classify subjects as AD patients or healthy individuals using the tool PALZ. The various methods of generating the rCBF method resulted in different PET_SCORES_ for the images and, therefore, distinct correlations with FDG scores and thresholds for classifying the subjects. The results presented here suggest that *R*_1_ parametric maps might be the best approach to generate rCBF images for diagnostic purposes provided that the threshold for classification is adjusted. Further research should focus on exploring how PET_SCORES_ correlate with disease progression in longitudinal studies.

## Additional files

Additional file 1: An overview of the steps taken for analysing images. (DOCX 20 kb)

Additional file 2: **Table S1.** Mean ± SD and range [minimum–maximum] of all methods per group. (DOCX 14 kb)

## Data Availability

The datasets generated and/or analysed during the current study are available with the corresponding author upon reasonable request.

## Abbreviations

AD: Alzheimer’s disease
Aβ: Amyloid-β
ePIB: Early-stage PIB
FDG: 2-[^18^F]-Fluoro-2-deoxy-d-glucose
FWHM: Full width and half maximum
HC: Healthy control
*k*_2_′: Efflux parameter of the reference region
MCI: Mild cognitive impairment
MNI: Montreal Neurological Institute
MRI: Magnetic resonance imaging
NIA-AA: National Institute on Aging Alzheimer’s Association
PALZ: PMOD Alzheimer’s Discrimination Tool
PET: Positron emission tomography
PIB: ^11^C-labelled Pittsburgh Compound B
*R*_1_: Ratio of tracer influx relative to the reference region
rCBF: Regional cerebral blood flow
ROC: Receiver operating characteristic
SRTM: Simplified reference tissue model
SRTM2: Simplified reference tissue model 2
SUVR: Standardized uptake value ratio
