# Supplementary material for: Diagnostic performance of regional cerebral blood flow images derived from dynamic PIB scans in Alzheimer’s disease
Source: EJNMMI Res. 2019 Jul 4;9:59. doi: 10.1186/s13550-019-0528-3 (PMC6609664; doi:10.1186/s13550-019-0528-3)
Supplement: Supplementary file 1 — An overview of the steps taken for analysing images. (DOCX 20 kb) [file 13550_2019_528_MOESM1_ESM.docx]

**Supplemental Material**

*PALZ Pipeline*

PALZ is a tool that implements the method developed by Herholz and colleagues (7) to aid the diagnosis of AD patients based on the metabolic pattern present on FDG PET scans. This tool has been tested and validated using a multicentre database. It starts by normalizing the provided image to the standard PET template provided by SPM99 software (Wellcome Trust Centre for Neuroimaging, UK), and then smoothes the images using a 12-mm Gaussian filter FWHM. The next step is to overlay a predefined mask (which contains regions from the brain that have the glucose metabolism preserved in AD, such as midbrain, putamen, insula, and sensorimotor and visual cortex), and normalize the voxel values within this mask to an average intensity of 1. Then, using a two-sample t-test, PALZ compares the values from the provided image to the values from a database of 49 HC from different centres, generating the t-values for each voxel. Next, PALZ sums the t-values of voxels with an age-adjusted *p* < 0.05 (uncorrected) within a pre-defined AD mask (temporoparietal cortex, posterior cingulate, precuneus, and frontal association cortex), resulting in the t-sum of the subject. This value is then compared to the upper 95% confidence limit of an independent database of healthy subjects (equals to 11089). Finally, the tool calculates a PET score for the image using the following equation:

$PET_{SCORE}=\log_{2} \left( \frac{t_{sum}}{11089}+1 \right)$.

The threshold of the *PET_SCORE_* for the subject to be classified as AD is 1. All HC are expected to present scores lower than the threshold.
