# Supplementary material for: Diagnostic performance of regional cerebral blood flow images derived from dynamic PIB scans in Alzheimer’s disease
Source: EJNMMI Res. 2019 Jul 4;9:59. doi: 10.1186/s13550-019-0528-3 (PMC6609664; doi:10.1186/s13550-019-0528-3)
Supplement: Supplementary file 2 — Table S1. Mean ± SD and range [minimum–maximum] of all methods per group. (DOCX 14 kb) [file 13550_2019_528_MOESM2_ESM.docx]

**Supplemental Table 1.** Mean ± SD and range [minimum – maximum] of all methods per group.

| **Group** |  | **FDG** | **R1** | **ePIB(20-130s)** | **ePIB(1-8min)** |
| --- | --- | --- | --- | --- | --- |
| **AD** | **Mean ± SD** | 2.50 ± 0.71 | 2.70 ± 0.73 | 2.93 ± 0.71 | 2.10 ± 0.77 |
|  | **Range** | [1.04 – 4.21] | [0.86 – 3.92] | [1.04 – 4.14] | [0.37 – 3.61] |
| **MCI+** | **Mean ± SD** | 1.42 ± 0.68 | 1.96 ± 0.55 | 2.19 ± 0.69 | 1.50 ± 0.51 |
|  | **Range** | [0.45 – 2.60] | [1.24 – 2.75] | [0.57 – 3.06] | [0.61 – 2.19] |
| **MCI-** | **Mean ± SD** | 1.25 ± 0.80 | 1.86 ± 0.67 | 2.20 ± 0.64 | 1.60 ± 0.60 |
|  | **Range** | [0.06 – 2.46] | [0.46 – 2.80] | [0.69 – 3.03] | [0.28 – 2.50] |
| **HC** | **Mean ± SD** | 0.64 ± 0.32 | 1.36 ± 0.41 | 1.61 ± 0.41 | 1.29 ± 0.32 |
|  | **Range** | [0.14 – 1.43] | [0.69 – 2.03] | [1.01 – 2.21] | [0.82 – 1.88] |
